# A Scoping Review of Adolescent Health Indicators

**DOI:** 10.1016/j.jadohealth.2021.04.026

**Published:** 2021-09

**Authors:** Holly Newby, Andrew D. Marsh, Ann-Beth Moller, Emmanuel Adebayo, Peter S. Azzopardi, Liliana Carvajal, Lucy Fagan, Howard S. Friedman, Mariame Guèye Ba, Ann Hagell, Alison Morgan, Elizabeth Saewyc, Regina Guthold

**Affiliations:** a Maternal, Newborn, Child and Adolescent Health and Ageing Department, WHO, Geneva, Switzerland; b UNDP/UNFPA/UNICEF/WHO/World Bank Special Programme of Research, Development and Research Training in Human Reproduction (HRP), Department of Sexual and Reproductive Health and Research, World Health Organization, Geneva, Switzerland; c Adolescent Health Unit, Institute of Child Health, University of Ibadan, Ibadan, Nigeria; d Global Adolescent Health Group, Burnet Institute, Melbourne, Victoria, Australia; e Adolescent Health and Well-being Program, Aboriginal Health Equity Theme, South Australian Health and Medical Research Institute, Adelaide, South Australia, Australia; f Centre for Adolescent Health, Department of Paediatrics, University of Melbourne, Melbourne, Victoria, Australia; g Division of Data Analytics Planning and Monitoring, Data and Analytics Section, UNICEF, New York, New York; h Department of Global Public Health, Karolinska Institutet, Stockholm, Sweden; i UN Major Group for Children and Youth, London, United Kingdom; j Imperial College Healthcare NHS Trust, London, United Kingdom; k Technical Division, United Nations Population Fund, New York, New York; l University Cheikh Anta Diop of Dakar, Faculty of Medicine, Pharmacy and Odontology/Gynecology, Dakar, Senegal; m Obstetrics Clinic, University Teaching Hospital A. Le Dantec, Dakar, Senegal; n Association for Young People's Health, London, United Kingdom; o Global Financing Facility, World Bank Group, Washington, District of Columbia; p Nossal Institute for Global Health, University of Melbourne, Melbourne, Victoria, Australia; q Stigma and Resilience Among Vulnerable Youth Centre, School of Nursing, University of British Columbia, Vancouver, British Columbia, Canada

**Keywords:** Adolescent, Adolescent health, Youth health, Adolescent health services, Global health, Health status indicators, Social determinants of health, Health behavior, Health risk behavior, Review

## Abstract

**Purpose:**

A host of recent initiatives relating to adolescent health have been accompanied by varying indicator recommendations, with little stakeholder coordination. We assessed currently included adolescent health–related indicators for their measurement focus, identified overlap across initiatives, and determined measurement gaps.

**Methods:**

We conducted a scoping review to map the existing indicator landscape as depicted by major measurement initiatives. We classified indicators as per 33 previously identified core adolescent health measurement areas across five domains and by age groups. We also identified indicators common across measurement initiatives even if differing in details.

**Results:**

We identified 413 indicators across 16 measurement initiatives, with most measuring health outcomes and conditions (162 [39%]) and health behaviors and risks (136 [33%]); followed by policies, programs, and laws (49 [12%]); health determinants (44 [11%]); and system performance and interventions (22 [5%]). Age specification was available for 221 (54%) indicators, with 51 (23%) focusing on the full adolescent age range (10–19 years), 1 (<1%) on 10–14 years, 27 (12%) on 15–19 years, and 142 (64%) on a broader age range including adolescents. No definitional information, such as numerator and denominator, was available for 138 indicators. We identified 236 distinct indicators after accounting for overlap.

**Conclusion:**

The adolescent health measurement landscape is vast and includes substantial variation among indicators purportedly assessing the same concept. Gaps persist in measuring systems performance and interventions; policies, programs, and laws; and younger adolescents' health. Addressing these gaps and harmonizing measurement is fundamental to improve program implementation and accountability for adolescent health globally.

**Implications and Contribution**

A large number of indicators are being used to measure adolescent health. Indicator definitions are inconsistent across different initiatives or lack detail, hampering use and comparability of the data. Measurement gaps need to be filled immediately to enable better tracking of adolescent health.

See Related Editorial on p.357

Adolescence has been recognized as a critical time for physical, mental, and behavioral development, creating a foundation for the rest of an individual's life [1,2]. Notwithstanding the importance of this period of life, there has been relatively little cross-country comparable tracking of the health of adolescents [3], which in turn has resulted in lack of attention and accountability. Even initiatives that encompass adolescent health often include only a few indicators that are commonly focused on specific themes such as sexual and reproductive health, not necessarily adapted for use in adolescents and often without harmonized names or definitions [4,5].

Indeed, the gap in adolescent-specific health measures is a result of a historical lack of attention to these crucial years. For example, widely used population-based household surveys such as the Demographic and Health Surveys and the Multiple Indicator Cluster Surveys [6] emerged out of a global prioritization of the health of young children and women of reproductive age (15–49 years). Although this information is valuable for specific purposes, it falls short of addressing a broader range of issues related to the lives and experiences of adolescents. Information on younger adolescents aged 10–14 years heavily relies on school-based surveys conducted across countries covering all income groups [7,8]; however, information on young adolescents not attending school – often including the most vulnerable population groups – is lacking globally.

Recent years have brought a host of new initiatives relating to adolescents' health. The Lancet Commission on Adolescent Health and Wellbeing [2], the Global Accelerated Action for the Health of Adolescents [1], the Global Strategy for Women's, Children's, and Adolescents' Health (2016–2030) [9], and Countdown to 2030 [10] all represent a critical step forward in emphasizing the significance of the adolescent period. The importance of measurement has been highlighted in these initiatives. The Lancet Commission, for example, emphasized the role of indicators in aligning response to needs and ensuring accountable actions. Each initiative, however, has proposed its own set of indicators for tracking progress.

These efforts, together with numerous others, address what has been perceived as a long-standing deficit of indicators related to adolescents globally [11] by highlighting critical topics and advancing adolescent-specific indicators such as those relating to life satisfaction and mental health. Although these developments have helped to advance the long-neglected adolescent measurement field, the increase in both proposed indicators and measurement initiatives, however, has come about with little coordination, as previously noted [12,13]. The result has been inconsistent across initiatives, including long-standing indicators, as well as a substantial number of new indicators, some of which are poorly defined and difficult to measure, whereas gaps in the topics addressed remain.

To strengthen coordination, technical standards, and capacity for adolescent health measurement, the World Health Organization (WHO), in collaboration with United Nations (UN) H6+ partner agencies (Joint UN Program on HIV/AIDS, UN Educational, Scientific and Cultural Organization, UN Population Fund, UN Children's Fund, UN Women, the World Bank Group, and the World Food Program), established the Global Action for Measurement of Adolescent Health (GAMA) Advisory Group, consisting of 17 global adolescent health experts [11,14]. A primary goal of the GAMA Advisory Group is to identify a set of priority adolescent health indicators, on which robust measurement standards will be built and harmonized guidance will be developed. Two work streams were determined as necessary first steps toward this goal: (1) identify measurement domains and identify core areas for adolescent health measurement globally [12] and (2) compile available indicators related to these core measurement areas. The purpose of this article is to map existing indicators for adolescent health, assess what they measure, and highlight overlap across initiatives and measurement gaps.

## Methods

### Overview of study design

We used a scoping review design [15,16] to map the existing indicator landscape as depicted by major measurement initiatives (Preferred Reporting Items for Systematic reviews and Meta-Analyses extension for scoping review checklist; see Supplementary Table 1). First, we reviewed 16 previously identified global and regional initiatives including adolescent health indicators (Table 1). Second, we compiled and categorized all indicators related to adolescents (defined by the WHO as individuals aged 10–19 years [17]), included in these initiatives. Third, we mapped the indicators as per the previously identified GAMA core adolescent health measurement areas. These steps, which build on work detailed in a previous publication [12], are summarized in the following text.

**Table 1:** Measurement initiatives and indicator compilations reviewed for adolescent health indicators

ID	Initiative name	Number of indicators included in mapping^q^
1	Global indicator framework for the Sustainable Development Goals and targets of the 2030 Agenda for Sustainable Development^a^	29
2	The Lancet Commission on Adolescent health and Well-being^b^	10
3	Indicator and Monitoring Framework for the Global Strategy for Women's, Children's, and Adolescents' Health (2016–2030) c	27
4	Countdown to 2030^d^	46
5	Family Planning 2020^e^	5
6	Adolescent Country Tracker^f^	18
7	Global Reference List of 100 Core Health Indicators^g^	33
8	Global Reference List of Health Indicators for Adolescents (aged 10–19 years)^h^	27
9	Core Indicators for Adolescent Health: A Regional Guide (EMRO)^i^	27
10	Commonwealth Youth Development Index^j^	9
11	INSPIRE Indicator Guidance and Results Framework^k^	46
12	Measurement of Mental Health among Adolescents at the Population Level initiative^l^	10
13	Monitoring and Evaluation Guidance for School Health Programs^m^	75
14	Measuring the Education Sector response to HIV and AIDS: guidelines for the construction and use of core indicators^n^	5
15	UNECE Monitoring Framework for the ICPD Program of Action beyond 2014^o^	27
16	WHO's 13th General Program of Work Impact Framework^p^	19

AIDS = Acquired Immunodeficiency Syndrome; EMRO = World Health Organization Regional Office for the Eastern Mediterranean; HIV = Human Immunodeficiency Virus; ICPD = International Conference on Population and Development; UNECE = United Nations Economic Commission for Europe; WHO = World Health Organization.

a United Nations. General Assembly. *A/RES/71/313. Global indicator framework for the Sustainable Development Goals and targets of the 2030 Agenda for Sustainable Development*. New York, USA: United Nations; 2017.

b Azzopardi PS, Hearps SJC, Francis KL, et al. Progress in adolescent health and wellbeing: tracking 12 headline indicators for 195 countries and territories, 1990-2016. *Lancet*. 2019; 393(10,176):1101-1118.

c Every Woman Every Child. *Indicator and Monitoring Framework for the Global Strategy for Women's, Children's and Adolescents' Health 2016-2030*. New York2016.

d Countdown to 2030: tracking progress towards universal coverage for reproductive, maternal, newborn, and child health. *Lancet*. 2018; 391(10,129):1538-1548.

e Family Planning 2020. FP 2020. 2018; https://www.familyplanning2020.org/. Accessed 4 December, 2019.

f UNICEF. Adolescent Country Tracker. 2018; https://data.unicef.org/resources/adolescent-country-tracker/. Accessed 4 December, 2018.

g World Health Organization. *Global Reference List of 100 Core Health Indicators (plus health-related SDGs)*. Geneva, Switzerland2018.

h World Health Organization. *Global Reference List of Health Indicators for Adolescents (aged 10-19 years)*. Geneva, Switzerland2015.

i World Health Organization ROftEM. *Core indicators for adolescent health: a regional guide*. Cairo, Egypt: World Health Organization. Regional Office for the Eastern Mediterranean; 2014.

j Commonwealth T. The Commonwealth Youth Development Index. 2016; https://thecommonwealth.org/youthdevelopmentindex. Accessed 7 April, 2020.

k United Nations Children's Fund. *INSPIRE Indicator Guidance and Results Framework - Ending Violence Against Children: How to define and measure change*. New York, USA: UNICEF; 2018.

l UNICEF. Measurement of Mental Health Among Adolescents at the Population Level (MMAP). 2020; https://data.unicef.org/topic/child-health/mental-health/mmap/. Accessed 2 July, 2020.

m UNESCO. *Monitoring and Evaluation Guidane for School Health Programs*. Paris, France: UNESCO; 2014.

n UNESCO. *Measuring the education sector response to HIV and AIDS. Guidelines for the construction and use of core indicators*. Paris, France: UNESCO; 2013.

o UNECE and UNFPA. *UNECE Monitoring Framework for the ICPD Programme of Action beyond 2014*. Geneva and Istanbul: UNECE and UNFPA; 2018.

p World Health Organization. WHO 13th General Programme of Work (GPW 13) Impact Framework: Targets and indicators. 2018; https://www.who.int/about/what-we-do/GPW13_WIF_Targets_and_Indicators_English.pdf. Accessed 7 April, 2020.

q Only those indicators pertaining to 33 core areas for adolescent health measurement identified by GAMA were included in the mapping.

### Initiatives reviewed

Adolescent health measurement initiatives were identified through expert consultations and a review of previous reports with GAMA Advisory Group members, UN partners, and WHO topic-specific and regional focal points for adolescent health. Initiatives were included if they (1) included recommendations about adolescent health measurement; (2) proposed at least one indicator specifically including “adolescent,” “youth,” or “young people,” or the entire or part of the adolescent age range 10–19 years; and (3) were intended to be used across multiple countries.

### Indicator inclusion criteria

We reviewed these initiatives, searching for any adolescent-related indicators (defined as summary measures of an outcome of interest [18]) pertaining to 33 core measurement areas identified for adolescent health measurement through an expert consensus process led by GAMA (Table 2). We were not restrictive in our approach but included any indicator that appeared to pertain both to adolescents and one of the 33 core measurement areas. Therefore, any indicator either overlapping with the adolescent age group (e.g., an indicator referring to women aged 15–49 years) or referring to “adolescents,” “young people,” or “youth” in the name or definition was compiled.

**Table 2:** Number of indicators included by domain and core measurement area

GAMA measurement domain and core adolescent health measurement area	Total indicators listed	Distinct indicators
	#	#
Social, cultural, economic, educational, and environmental determinants of health	44	24
Population (total and % adolescents)	4	2
Education level/schooling status	21	11
Income level and poverty	11	6
Gender	8	5
Health behaviors and risks	136	70
Weight status	14	6
Alcohol use	13	5
Substance use (other than alcohol and tobacco)	8	5
Tobacco use	9	2
Dietary behavior	16	15
Physical activity	13	8
Bullying	4	2
Sexual health; reproductive health; and contraception^a^	59	27
Policies, programs, and laws	49	45
Adolescent health policies/plans (availability, implementation, funding, and M&E)	36	35
Adolescent health protective laws (availability, implementation, funding, and M&E)	13	10
Systems performance and interventions	22	20
Health service availability and access	12	12
Health service quality	1	1
Immunization	4	2
System for monitoring and surveillance of adolescent health	5	5
Health outcomes and conditions	162	77
All-cause mortality	6	1
Cause-specific mortality	39	14
HIV/AIDS and STIs excluding HIV/AIDS^a^	38	19
Self-harm	2	2
Anxiety disorders and depressive disorders^a^	15	11
Disability	0	0
Road injury	4	4
Interpersonal violence, sexual violence, and gender-based violencea	44	24
Adolescent fertility	14	2
Total indicators	413	236

GAMA = Global Action for Measurement of Adolescent Health; M&E = monitoring and evaluation; HIV/AIDS = human immunodeficiency virus/acquired immune deficiency syndrome; STI = sexually transmitted infection.

a Separate core measurement areas have been combined owing to the numerous indicators that were cross listed.

### Compilation and classification of indicators

For each indicator, relevant metadata (defined as information about the indicators [19]) were extracted in a spreadsheet. The spreadsheet included details to (1) understand how the indicators are defined and measured and (2) organize and categorize indicators to facilitate analysis, based on earlier, similar work [20] (Panel 1). The captured details were based on the readily available metadata in the key reference for the initiative. Notably, all indicators meeting the inclusion criteria were compiled as presented by the initiative's documentation. In this initial compilation, every indicator was listed as a unique entry, even if indicators from different initiatives were similar.

Panel 1 **Structure of GAMA indicator mapping.**

• Measurement domain
• Core measurement area
• Indicator name
• Definition
• Numerator
• Denominator
• Indicator type: inputs and processes, output, outcome, and impact
• Broad age groupings: 10–14, 15–19, or 10–19 years
• Age range (indicator specific)
• Status of indicator: in use or aspirational
• Feasible data sources
• Measurement initiative including the indicator
• Distinct indicators

We developed a classification framework to group and examine the indicators in several different ways.

First, we ensured that each indicator was categorized as per the 33 previously identified core measurement areas under five broad domains. Numerous indicators encompassed more than one core measurement area. In these cases, we cross-referenced all applicable core measurement areas. “Human papillomavirus vaccine coverage among adolescents,” for example, was categorized under immunization as the primary core measurement area but was cross-referenced under sexually transmitted infections (excluding HIV/AIDS). To avoid double counting of indicators in our analysis, we grouped several of the core measurement areas together (Table 2). Furthermore, we decided that any indicator pertaining to a policy, program, or law would be categorized in the appropriate core measurement area under the domain policies, programs, and laws. However, if the indicator was also related to a health behavior or a health outcome (such as a policy on HIV/AIDS), it was cross-listed with the appropriate core measurement area under these domains.

Second, we classified each indicator in accordance with the age group based on the accompanying definitional information and then labeled it in accordance with three key parameters. The first parameter refers to broad age groupings, capturing whether the indicator pertains to adolescent ages 10–14 and 15–19 years or the entire adolescent age range of 10–19 years. The second parameter captures whether the indicator is intended to measure the entire specified age range. For example, an indicator that is based on a denominator of adolescents aged 13–17 years would be classified as pertaining to the broad age range of 10–19 years but would be additionally labeled as not covering the entire age range. The third parameter reflects whether the indicator's specified age range extends beyond the adolescent years. This was a common feature of many indicators that are based on a population of reproductive ages 15–49 years, although the data generally allow for disaggregation by five-year age groups. There are also indicators referring to “children” younger than 18 years of age.

Finally, we attempted to identify indicators cited by more than one initiative, even if they appeared to differ in measurement details (referred to as “overlapping indicators” herein). By identifying indicators listed more than once, we were able to distinguish how many distinctive indicators the compilation contained, as opposed to the total number of separately listed indicators, which included numerous overlapping indicators intending to measure the same concept. However, even when indicators appear to refer to the same concept, available metadata can suggest substantial variation including age range, total population versus school-based population, sex, and the reference period (time frame). The approach we took to identify distinct versus overlapping indicators is presented in Panel 2, along with examples.

Panel 2 **Summary of approach for determining distinct versus overlapping indicators**

**Table:** 

Parameter	Approach	Example
Age	Unless there was a clear intention to measure younger versus older adolescents, indicators referring to differing age ranges were considered to be overlapping.	The compilation includes 12 separately listed “adolescent birth rate” indicators and although the age specifics varied, we considered them all to pertain to the same underlying measurement concept.
School-based versus total populations	Indicators appearing to measure the same concept but just for school populations versus total populations were considered overlapping.	The indicator “current alcohol use among adolescents,” which is defined as “proportion of adolescents who had at least one alcoholic drink on one or more days during the past 30 days” was considered to be overlapping with “percentage of students who had at least one alcoholic drink during the last 30 days,”
Gender	In most cases, we considered an indicator with a female-only denominator to overlap with an indicator encompassing both women and men. There were some exceptions to this, particularly if other definitional details or descriptions in the metadata made it clear that the indicator had a gender-specific purpose.	Of eight indicators on intimate partner violence, for example, six referred to women only, one referred to women and men, and one did not provide definitional information related to sex. We considered these indicators to be overlapping.
Time frame	Most indicators did not include a specific reference time period and some did appear to be operationalizing the same concept in differing ways. There are some indicators, however, where the time element provided a critical difference in the measurement concept and these indicators were considered distinct.	“Percentage of students aged 13–15 years who have ever tried cannabis” captures cumulative lifetime experience and was thus considered distinct from indicators measuring current use such as “percentage of students who have used marijuana during the past 30 days” and “current cannabis use among adolescents” (defined as “proportion of adolescents who report use of cannabis in the past 30 days”), which we also considered overlapping.

Decisions regarding indicators lacking metadata were based solely on the indicator name. For example, the indicator “adolescent population” as referred to by one initiative was not accompanied by any definitional information, and so, for the purpose of identifying correspondent indicators from other initiatives, it was assumed to overlap with “total adolescent population” as opposed to “percentage of adolescent population (10–19 years).”

## Results

The compilation comprised a total of 413 separately listed indicators identified across 16 initiatives, including 44 (11%) categorized as primarily relating to the domain determinants of health, 136 (33%) relating to health behaviors and risks, 49 (12%) categorized under policies, programs, and laws, 22 (5%) under systems performance and interventions, and 162 (39%) primarily pertaining to health outcomes and conditions [21]. Indicators spanned 32 of the 33 core measurement areas as previously identified by GAMA, with no indicator pertaining to the core measurement area disability (Table 2).

The majority of the reviewed measurement initiatives covered multiple topical areas, and yet, none encompassed more than two-thirds of the 33 GAMA core adolescent health measurement areas (Table 3). The core measurement areas commonly addressed across initiatives were HIV/AIDS and adolescent fertility rate (represented by 12 initiatives each) as well as weight status, reproductive health, cause-specific mortality, and interpersonal violence (represented by 11 initiatives each). On the other hand, numerous core measurement areas were addressed by fewer than five initiatives, including income level and poverty, bullying, and immunization (represented by four initiatives each); population, system for monitoring and surveillance of adolescent health, and anxiety disorders (represented by three initiatives each); health service quality (represented by two initiatives); and disability, which no initiative addressed.

**Table 3:** GAMA core measurement areas covered by reviewed initiatives

Initiative/Measurement area	Core 100	Global strategy	Countdown to 2030	SDGs	Global reference list of health indicators for adolescents	Adolescent country tracker	Inspire	Lancet commission	Global youth development Index	UNECE monitoring framework (ICPD)	FP2020	Measuring the education sector response to HIV and AIDS	EMRO core indicators	FRESH	WHO GPW	MMAP	Initiatives covering area (primary or cross-listed)
Social, cultural, economic, educational, and environmental determinants of health																	
Population (total and % adolescents)																	3
Education level/schooling status																	9
Income level and poverty																	4
Gender																	9
Health behaviors and risks																	
Weight status																	11
Alcohol use																	10
Substance use (other than alcohol and tobacco)																	6
Tobacco use																	9
Dietary behavior																	7
Physical activity																	7
Bullying																	4
Sexual health																	10
Reproductive health																	11
Contraception																	10
Policies, programs, and laws																	
ADH policies/plans (availability, implementation, funding, and M&E)																	5
ADH protective laws (availability, implementation, funding, and M&E)																	5
Systems performance and interventions																	
Health service availability and access																	5
Health service quality																	2
Immunization																	4
System for monitoring and surveillance of ADH																	3
Health outcomes and conditions																	
All-cause mortality																	6
Cause-specific mortality																	11
HIV/AIDS																	12
STIs (excluding HIV/AIDS)																	9
Self-harm																	9
Anxiety disorders																	3
Depressive disorders																	8
Disability																	0
Road injury																	6
Interpersonal violence																	11
Sexual violence																	10
Gender-based violence																	9
Adolescent fertility																	12
Total areas represented within each initiative	20	20	19	22	21	14	20	10	8	20	2	4	18	19	20	3	

Dark blue = The initiative includes one or more indicators with a primary focus on the listed measurement area. Light blue = The initiative includes no indicators with a primary focus on the listed measurement area but includes one or more indicators that are cross-listed with this area. Abbreviations: ADH = Adolescent health; GAMA = Global Action for Measurement of Adolescent Health; M&E = Monitoring and evaluation; STI = sexually transmitted infection.

Analysis in accordance with the age group was hampered by insufficient details in the underlying metadata. The compilation included a total of 352 entries based on individuals as the unit of measurement (e.g., as opposed to schools or policies ). Based on available information, we were able to classify only 221 indicators by age, which is less than two-thirds. Thus, although it was clear from the initiative or the indicator name that the indicator was related to the adolescent age range, we were unable to classify 126 individual-level indicators as pertaining to a specific age group because the underlying metadata did not include sufficient information regarding age.

**Figure 1:**
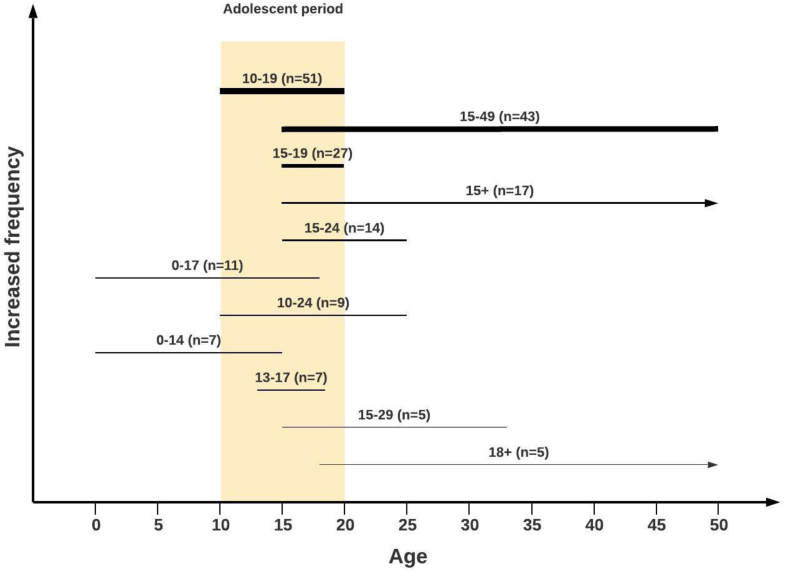
Age coverage across adolescent health indicators. Note: This figure presents the 11 most common age ranges covered by indicators in the GAMA mapping. The figure presents all age ranges that occurred at least five times, accounting for 196 of the 221 indicators (87%) where a specific age range was provided. The ordering and width of each line present the relative frequency with which the age range occurred (least common = thinnest line, bottom of figure; most common = thickest line, top of figure). GAMA, Global Action for Measurement of Adolescent Health

For those 221 indicators that we were able to classify by age, the analysis revealed substantial variation in the age ranges different indicators were intended to measure. Less than one-quarter – 51 indicators – pertained specifically to the full adolescent age group of 10–19 years. In addition, just one indicator was specific to only the entire young adolescent age range (10–14 years) and 27 captured the entire older adolescent age range (15–19 years). Beyond the indicators pertaining to specific adolescent age groups, many indicators encompassed years well beyond the adolescent age range. Notably, 43 indicators were based on ages 15–49 years, reflecting the reality that many indicators suggested for older adolescents were derived from measures mainly focusing on individuals of reproductive age. Similarly, there were indicators overlapping with adolescence that were intended to measure aspects of children's lives, such as the 11 indicators based on ages 0–17 years (Figure 1).

After identifying overlapping indicators, a subset of 236 indicators of the total 413 (57%) were deemed distinct entries. Most core measurement areas contained numerous overlapping indicators, demonstrating that two or more initiatives had identified identical, or at least similar, indicators. There were some notable exceptions, however, such as those core measurement areas falling under the policies, programs, law domain, and the systems performance and intervention domain, which contained indicators that, even if related to some extent, were nonetheless different enough to be considered distinct. The core measurement areas of dietary behavior and road injury were similarly characterized by a lack of overlapping indicators across the initiatives.

## Discussion

Increased focus on adolescent health in recent years has led to welcome advances in the development, promotion, and use of indicators related to this population group, as evidenced by the number of measurement initiatives included in this review. On the other hand, the sheer number of indicators being promoted by these initiatives is overwhelming and impossible to track in a meaningful way. Although many of the separately listed 413 indicators in our review were overlapping, the 236 indicators that were deemed distinct still represent a huge monitoring burden. This finding underscores the need for prioritization, which is one of GAMA's primary goals [14].

Our scoping review revealed deficiencies, gaps, and substantial variations across measurement initiatives' metadata. For instance, specific information regarding age was provided for less than two-thirds of the separately listed individual-based indicators in the compilation, and where information on age was provided, it often varied across indicators. The adolescent birth rate provides an illustrative example of the challenges: of the 12 separately listed indicators, five referred only to ages 15–19 years; 1 referred to ages 10–14 years; three referred to both ages 10–14 and 15–19 years; two were internally inconsistent, meaning the age-pertinent details in the name and definition were not aligned; and 1 indicator was simply named “adolescent birth rate” and lacked any definitional information. This is not only confusing but can also result in inconsistent measurement and hinder comparability. To resolve these issues, work on standardizing age groups in data collection, analysis, and reporting of health data across the life course is underway. This work is led by the WHO and supported not only by GAMA but also by similar advisory groups for other periods of the life course [20,22]. Furthermore, in developing detailed and harmonized measurement guidance for selected priority indicators, GAMA aims at improving consistency in measurement of the most relevant adolescent health indicators [14].

This scoping review also demonstrated that measurement in some of GAMA's previously defined domains and core areas is more advanced than in others. The policies, programs, laws domain, and the systems performance and interventions domain, for example, lack long-established, standardized indicators, and thus, there was little convergence in the indicators recommended by different initiatives. Notably, measurement of policies and systems has long lagged behind health outcomes and conditions or even health behaviors and risks, measurement of which has often been captured in both routine health information systems as well as population-based surveys [23]. As adolescent health measurement advances, it will be important to give special consideration to areas where measurement has historically lagged to ensure proper coordination of relevant tasks and conduction of necessary research as a basis for indicator development.

However, measurement domains and areas that are more advanced and contain a large number of sometimes long-standing indicators bring different challenges. Our scoping review suggests that in at least some cases, the inclusion of indicators in initiatives was driven more by the supply of existing indicators – frequently those that are easy to measure – than demand for measurement specific to adolescence. For example, adolescent health indicators in the areas of sexual and reproductive health and contraception, HIV/AIDS, and sexually transmitted infections are characterized by an imbalance: numerous indicators currently in widespread use are simply the disaggregation of those based primarily on women of reproductive age 15–49 years, whereas there are only few indicators pertaining to younger adolescents aged 10–14 years. Similarly, in other areas, indicators that are used in adolescent health may be derived from those originally developed for children younger than 18 years of age and not for adolescents specifically [18]. Although an indicator originally developed for a broader age group may be perfectly fit for adolescent measurement, this is a point that warrants consideration both in terms of the indicator itself and its operationalization. Generally, in areas where measurement is advanced, there is often a need to review indicators, ensuring they are adequate to capture the adolescent experience, and then to harmonize methodologies, important tasks that GAMA will pay special attention to as its work is being advanced.

Several important measurement gaps were revealed through our scoping review. Interestingly, they were not limited to domains and areas lagging behind in measurement but were also present in areas containing a large number of long-standing indicators. This finding is consistent with a recent review showing that more than 800 indicators including variations of supposedly the same indicator were used in adolescent reproductive health [13], but there were still data deficiencies for unmarried youth, adolescent boys, and very young adolescents and in specific areas such as abortion, nonheterosexual behavior, or fertility intentions. Core measurement areas that appear to lack a sufficient number of indicators to broadly reflect the main concepts include, for instance, substance use other than alcohol and tobacco, health service quality, and, in particular, disability, for which none of the initiatives included an indicator. Furthermore, we also found a general lack of indicators for young adolescents and those not attending school [18].

The identified measurement gaps need to be filled immediately, understanding that gaps in indicator availability may be driven by a variety of factors. In certain cases, gaps may exist owing to long-standing measurement practices. For example, much available adolescent data are commonly collected in household surveys that focus on interviewing women aged 15–49 years, and thus data pertaining to women aged 10–14 years are not collected. Another common source of data is school-based surveys, which miss the out-of-school population. Complexity of measurement can present a different sort of methodological challenge resulting in a gap: robust measurement of disability, for example, requires a lengthy set of questions. Finally, the long-standing deficit of attention given to adolescent health means that some topics require more methodological work to develop robust indicators and measurement approaches. Unless these gaps can be systematically addressed, program managers and policy makers will necessarily base decisions on a partial – and potentially biased – picture of the adolescent population. The way forward must include innovative thinking around different options for collecting data on all adolescents – for example, stand-alone surveys or specialized questionnaires integrated into existing questionnaires [24] – and validation of existing indicators together with methodological work to develop new measurement approaches.

This study highlighted numerous challenges in trying to compile and classify indicators in a standardized way, and there are limitations to our results. First, given imprecise, contradictory, or missing metadata, categorization of indicators is necessarily imperfect. Repeated listing of what may have initially appeared to be the same indicator resulted in numerous differences between entries, starting with inconsistency in the name and, importantly, the specified age groups. This was true even for Sustainable Development Goals indicators [25], which are supported by detailed metadata and we originally assumed would be standardized across initiatives. Second, it was sometimes difficult to fully understand what the indicator was intended to measure based on the information provided by the initiative. In cases where no definitional information was provided, our classification had to solely rely on the indicator names, which varied greatly in detail. Thus, identifying overlapping indicators – those that are either true duplicates or have the same measurement intent but varying slightly in terms of the name and/or definition – was an inherently fraught exercise, although one we still felt was crucial to undertake. Entering all indicators into the compilation as we did, instead of trying to reconcile and synthesize during compilation, has the important advantage that differing definitions and operationalization for the same measurement concept can be reviewed and considered during the selection of priority indicators, which will in turn result in a more robust set of measures and guidance.

Third, this indicator compilation reflects a subset of existing indicators – those that were contained within the initiatives included in this scoping review – and thus presents a partial picture of the measurement landscape at a particular point of time. We identified initiatives via expert consultation and did not conduct a full search of the literature or include any initiatives at the country level. These global-level measurement initiatives may not reflect the full range of measures that are available. Notably, there are rapid developments in the field, evidenced by the recently debuted, United Nations International Children’s Emergency Fund–led Measurement of Mental Health among Adolescents at the Population Level initiative [26], which was subsequently added to our initial list of 15 initiatives. Furthermore, in terms of topical scope, we only considered 33 previously defined core measurement areas. Although this may have skewed our findings, it enabled us to focus our work. Notably, the 33 measurement areas were systematically identified by adolescent health experts considering not only the adolescent mortality and morbidity burden [12] but also input from young people and countries and we therefore believe that our mapping indeed includes the most relevant adolescent health issues.

This indicator compilation serves as an important basis for GAMA's continued work of reviewing existing indicator recommendations, systematically selecting priority indicators for each core measurement area, and ensuring that each selected priority indicator is supported by clear, consistent, and comprehensive metadata. This indicator selection process is currently underway and draws on existing guidance [18,[27], [28], [29]]. As part of this work, measurement gaps are being identified and will be put forward for further research, including exploring any potential barriers to the widespread adoption of these indicators. The GAMA priority indicators will then be promoted for use in countries and regularly revisited as new evidence emerges and as measurement gaps are filled. This long-term process will require continued investment in advocacy and careful consideration of both existing measurement systems and the country-specific contextual factors. To that end, the selection of GAMA priority indicators is undertaken in collaboration with a broad range of stakeholders, including UN H6+ partnership agencies, key measurement groups, country-level implementing partners, and the public.

The adolescent health measurement landscape is vast and includes substantial variation among indicators purportedly assessing the same concept. Important gaps persist, with a lack of robust, standardized indicators measuring systems performance and interventions, policies and laws, and the needs of men, those with disabilities, and younger adolescents, specifically. Addressing these gaps and harmonizing measurement approaches will be fundamental to improving policy and program implementation as well as accountability for adolescent health globally. Margaret Chan, the former Director-General of the WHO, has said repeatedly: “what gets measured gets done.” Consistent measurement is the first step to understanding the needs of adolescents across countries and over time, thus providing policy makers with actionable information that will enable them to improve the health of adolescents in their countries.
